# Joint associations of device-measured step count and sleep duration with incident major adverse cardiovascular events: prospective analysis of the UK Biobank

**DOI:** 10.1016/j.eclinm.2026.103769

**Published:** 2026-01-29

**Authors:** Jason Yun, Laura Brocklebank, Charlie Harper, Aiden Doherty

**Affiliations:** Big Data Institute, Nuffield Department of Population Health, University of Oxford, Old Road Campus, OX3 7LF, UK

**Keywords:** Accelerometry, Machine learning, Cardiovascular disease, Step count, Sleep duration, UK Biobank

## Abstract

**Background:**

The interaction between physical activity and sleep with cardiovascular disease remains poorly understood, despite both being key risk factors. This study investigated the independent and joint associations of device-measured step count and sleep duration with incident major adverse cardiovascular events (MACE).

**Methods:**

Prospective analysis of UK Biobank participants who wore a wrist-based accelerometer for seven days between 2013 and 2015. Open-source machine learning algorithms derived daily step count and overnight sleep duration. The outcome was incident MACE (cardiovascular death, non-fatal myocardial infarction or stroke, or revascularisation procedure), identified through electronic health record linkage. Cox proportional hazards models were used to examine independent and joint associations of median daily step count (low [<7500], intermediate [7500–11,000], high [>11,000]) and median overnight sleep duration (short [<6.5 h], intermediate [6.5–7.5 h], long [>7.5 h]) with incident MACE.

**Findings:**

Among 88,012 participants (mean age 62.2 years [standard deviation, SD 7.8]), 3817 were diagnosed with MACE during follow-up (median 7.9 years [interquartile range, IQR 7.3–8.4]). Low step count and short sleep duration were independently associated with a higher risk of MACE, but there was no evidence of an interaction between step count and sleep duration (*P* for interaction = 0.42). Compared with the reference group—participants with high step count and intermediate sleep duration—the highest risk of MACE was observed in participants with both low step count and short sleep duration (hazard ratio, HR: 1.84, 95% CI: 1.62–2.10, *p* < 0.0001).

**Interpretation:**

The results of this study show that higher daily step count does not fully attenuate the higher risk of cardiovascular disease associated with short sleep duration, reinforcing the importance of sufficient levels of both daily step count and sleep for the prevention of cardiovascular disease.

**Funding:**

10.13039/100010269Wellcome Trust (223100/Z/21/Z).

**Research in context**

**Evidence before this study**

We searched the Medline and Embase databases in June 2025 for articles published in English from database inception to June 18th, 2025. Key search terms included ‘physical activity’, ‘sleep’, and ‘cardiovascular disease’, along with their synonyms, using both MeSH and free-text terms (Table S1). Previous studies have reported mixed evidence for an interaction between physical activity and sleep duration with incident cardiovascular mortality (Table S2 and S3). Some found that higher levels of physical activity attenuated the higher risk associated with abnormal sleep duration, whereas others did not replicate these findings. Many previous studies relied on self-reported measures of physical activity and sleep duration, and reported physical activity metrics that are not easily interpretable. Moreover, by focussing exclusively on cardiovascular mortality, they included relatively few events.

**Added value of this study**

To our knowledge, this prospective cohort study is the first to investigate the joint associations of step count and sleep duration with incident major adverse cardiovascular events. This study used accelerometer data from over 88,000 UK Biobank participants, with the outcome identified through electronic health record linkage. We adjusted for a broad range of key covariates and addressed potential reverse causation by excluding participants with latent or prevalent cardiovascular disease. Although both fewer daily steps and short sleep duration were independently associated with a higher risk of major adverse cardiovascular events, we found no evidence of an interaction between step count and sleep duration.

**Implications of all the available evidence**

Physical activity and sleep are essential components of daily life. They are closely linked within the 24-h cycle and are both associated with cardiovascular disease. The results of this study show that more daily steps do not fully attenuate the higher risk of cardiovascular disease associated with short sleep duration, reinforcing the importance of sufficient levels of both daily steps and sleep for the prevention of cardiovascular disease.

## Introduction

Physical activity (PA) and sleep are essential components of daily life, both of which are associated with cardiovascular disease (CVD).^1^^,^^2^ Large prospective cohort studies have shown that meeting the World Health Organisation (WHO) recommendation of ≥150 min of moderate-intensity aerobic PA per week is associated with a 23% lower risk of CVD mortality.^3^ Sleep duration has been shown to have a J-shaped association with CVD, with both short and long durations linked to higher risk.^4^

PA and sleep are interrelated, with regular PA associated with improved sleep duration and quality.^5^ Furthermore, emerging evidence suggests that higher levels of PA may fully attenuate the higher risk of CVD associated with suboptimal sleep duration.^6^^,^^7^ However, this finding has not been replicated in other large prospective cohort studies, resulting in limited understanding of the potential joint associations of PA and sleep with CVD.^8^^,^^9^

A major limitation of the current literature is the reliance on self-report questionnaires, which are subject to recall and social desirability biases,^10^ and have been shown to correlate poorly with device-based measures such as polysomnography for sleep and accelerometry for PA.^11^^,^^12^ Several studies were statistically underpowered to detect a potential interaction, having ≤1000 events (Table S2 and S3). Most studies focused exclusively on CVD mortality, limiting understanding of the potential joint associations of PA and sleep with non-fatal cardiovascular events—an important consideration when developing primary prevention strategies. Finally, the use of complex PA metrics, such as metabolic equivalent of task (MET), may hinder the interpretation of findings in a public health context. Similar to general PA, higher daily step count is associated with a lower risk of CVD; however, step count is simpler to communicate, interpret, and measure.^13^

Therefore, this study aimed to investigate the independent and joint associations of accelerometer-measured step count and sleep duration with incident major adverse cardiovascular events (MACE) in over 88,000 participants enrolled in the UK Biobank.

## Methods

### Study design, participants, and procedures

This study used data from the UK Biobank, a large prospective cohort study of over 500,000 participants recruited between 2006 and 2010 from across the United Kingdom (excluding Northern Ireland).^14^ Between 2013 and 2015, a subset of 236,519 participants were invited to wear an Axivity AX3 triaxial accelerometer on their dominant wrist continuously for seven days to measure 24-h movement behaviours, including PA, sedentary behaviour, and sleep.^15^ Of those invited, 106,053 participants agreed to wear the device, and 103,712 provided useable raw acceleration data.

### Step count and sleep duration measurements

Step count and sleep duration were derived by applying two open-source machine learning algorithms to the raw acceleration data. These models were developed and evaluated by the Oxford Wearables Group.^16^^,^^17^ The step count model was evaluated against foot-facing video camera observation in 69 adults across one free-living and one laboratory study,^16^ while the sleep duration model was evaluated against polysomnography in 1166 adults across five laboratory studies.^17^

To derive step count, a hybrid self-supervised machine learning and peak detection algorithm (github.com/OxWearables/stepcount, version 3.7.0) was used, as previously described by Small et al.^16^ Participants were excluded if they did not have sufficient wear time, defined as ≥3 days of data with coverage in every 1-h period of the 24-h cycle. Non-wear time was defined as unbroken periods of ≥90 min during which the SD of acceleration on each axis was <13 mg. To address potential diurnal bias in wear time, recording interruptions and non-wear periods were imputed using the average value for the corresponding minute of the day across the remaining valid days.^18^ The primary exposure was median daily step count. Peak 30-min cadence (a measure of stepping intensity, defined as steps per minute) was calculated by averaging the 30 highest 1-min cadence values per day, then taking the mean across all days in the measurement period.^19^

To derive sleep duration, a self-supervised deep recurrent neural network algorithm (github.com/OxWearables/asleep, version 0.4.12) was used, as previously described by Yuan et al.^17^ Participants were excluded if they did not have sufficient wear time, defined as ≥22 h per day for ≥3 days (including ≥1 weekend day). Non-wear time was defined identically to the step count algorithm. The primary exposure was median overnight sleep duration, calculated across all noon-to-noon intervals during the measurement period. Sleep efficiency was calculated by dividing overnight sleep duration by the total time spent in bed each day, then averaging this ratio across all valid days.

After excluding participants with insufficient wear time, further exclusions were made if the device could not be calibrated, more than 1% of readings were ‘clipped’ (fell outside ±2 *g*) before or after calibration, average acceleration was implausibly high (>100 mg), or step count or sleep duration could not be estimated.

Time spent in 24-h movement behaviours—including moderate-to-vigorous physical activity (minutes/day), light physical activity (hours/day), total sedentary time (hours/day), and time in bed (hours/day)—were derived by applying a separate machine learning algorithm to the raw acceleration data (https://github.com/OxWearables/biobankAccelerometerAnalysis, v7.1.1), as previously described by Walmsley et al.^20^

### Outcome ascertainment

The primary outcome was the first occurrence of a MACE event, defined as death from any cardiovascular cause, non-fatal myocardial infarction or stroke, or revascularisation procedure. MACE events were identified through linkage to National Health Service (NHS) Digital for England, Secure Anonymised Information Linkage (SAIL) Databank for Wales, and the NHS Central Register for Scotland (Table S4). To identify incident cases of MACE, participants with a prior self-reported or hospital-recorded diagnosis were excluded from the analysis.

### Covariates

Potential confounders and mediators were selected a priori using a causal diagram based on the current literature (Figure S1). Demographic, socioeconomic, and lifestyle factors—including age, sex, ethnicity, Townsend Deprivation Index (TDI), smoking status, alcohol intake, and processed meat intake—were treated as confounders and derived from self-report (Table S5). Adiposity and cardiometabolic factors—including body mass index (BMI), glycated haemoglobin (HbA1c), blood pressure, and total cholesterol—were considered potential mediators and derived from physical measurements or blood samples (Table S5).

### Statistical analysis

To ensure adequate group sizes and improve the interpretability of our results, median daily step count was initially divided into tertiles and then reclassified by rounding the boundaries to the nearest 500 steps (low [<7500], intermediate [7500–11,000], high [>11,000]; Figure S2). Median overnight sleep duration was categorised similarly, with tertile boundaries rounded to the nearest 0.5 h (short [<6.5 h], intermediate [6.5–7.5 h], long [>7.5 h]; Figure S3). High step count and intermediate sleep duration were the reference groups. This approach was adopted because there are currently no universally accepted cut-points for device-measured step count or sleep duration, as existing public health guidelines are predominantly based on self-reported data.^21^^,^^22^

Baseline participant characteristics were reported as mean and standard deviation (SD) for normally distributed continuous variables, median and interquartile range (IQR) for non-normally distributed continuous variables, and number of participants and percentage (%) for categorical variables. Univariable associations between each covariate and the exposures (step count and sleep duration) were assessed using the Pearson χ^2^ test for categorical covariates, the one-way Analysis of Variance (ANOVA) test for normally distributed continuous covariates, and the Kruskal–Wallis test for non-normally distributed continuous covariates. Associations between each covariate and the outcome (incident MACE) were assessed using univariable Cox proportional hazards models. Analyses were performed on a complete-case basis; participants with missing data on the exposures, outcome, or confounders were excluded from the main analyses, and those with missing data on the potential mediators were excluded from the mediator-specific sensitivity analyses.

Multivariable Cox proportional hazards models were employed to examine the independent associations of step count and sleep duration with incident MACE,^23^ using age as the timescale consistent with previous studies.^16^^,^^17^ The proportional hazards assumption was assessed using Schoenfeld residuals; sex was the only variable to statistically violate the assumption, so all models were stratified by sex. Sequential adjustments for potential confounders were performed in the following order: ethnicity, education, TDI, smoking status, alcohol intake, processed meat intake, and finally step count or sleep duration (depending on the exposure). Both minimally adjusted and maximally adjusted hazard ratios (HRs) with their respective 95% confidence intervals (CIs) were reported in the main text. Floating absolute risks were also calculated to provide risk estimates and 95% CIs for each group independently, allowing direct comparisons between groups. These were used where appropriate to display 95% CIs in the figures.^24^ In addition to the maximally adjusted model, we also ran models with each potential mediator added sequentially to examine their impact on the HRs: BMI, HbA1c, blood pressure, and total cholesterol. These results are descriptive and should not be interpreted as formal evidence of mediation.

Participants were stratified into nine mutually exclusive groups based on their median daily step count and median overnight sleep duration to explore their combined effects on incident MACE (Table S6). Those with high step count and intermediate sleep duration served as the reference group. To formally test for an interaction, we performed a likelihood ratio (LR) test comparing the maximally adjusted model with and without an interaction term between step count and sleep duration.

### Sensitivity analysis

To examine potential reverse causation, participants diagnosed with MACE within the first two, four, and six years of follow-up were sequentially excluded. Additional analyses excluded participants with a prior self-reported or hospital-recorded diagnosis of any CVD or cancer, identified using the relevant medical classification codes (Table S4). To assess the robustness of the observed associations to competing risks, we performed cause-specific Cox regression treating MACE as the event of interest and non-CVD death as a competing risk, censoring individuals at the time of non-CVD death (N: 2161 without prior MACE). To assess the impact of sleep duration misclassification, participants with factors that could disrupt sleep were excluded, including self-reported shift workers, those with a prior diagnosis of sleep apnoea or restless leg syndrome, and those whose accelerometer wear period overlapped with daylight saving time changes. Adjustments for sleep efficiency and step cadence were performed to assess the potential confounding effects of sleep quality and stepping intensity on the observed associations. Finally, in a separate model, adjustments for sedentary time and time spent in moderate-to-vigorous physical activity were performed to assess potential confounding by inactivity and overall activity intensity.

All analyses were conducted using STATA (version 18.0), and evidence of suggestive statistical significance was defined as *p* < 0.05. Results have been reported according to the STROBE guidelines (Table S7).^25^

### Ethics statement

The UK Biobank received ethical approval by the NHS North West Multi-centre Research Ethics Committee (11/NW/0382). Participants gave informed consent to participate in the study before taking part.

### Role of the funding source

The funder of the study had no role in study design, data collection, data formal analysis, data interpretation, or writing of the report.

## Results

### Baseline characteristics

**Fig. 1:**
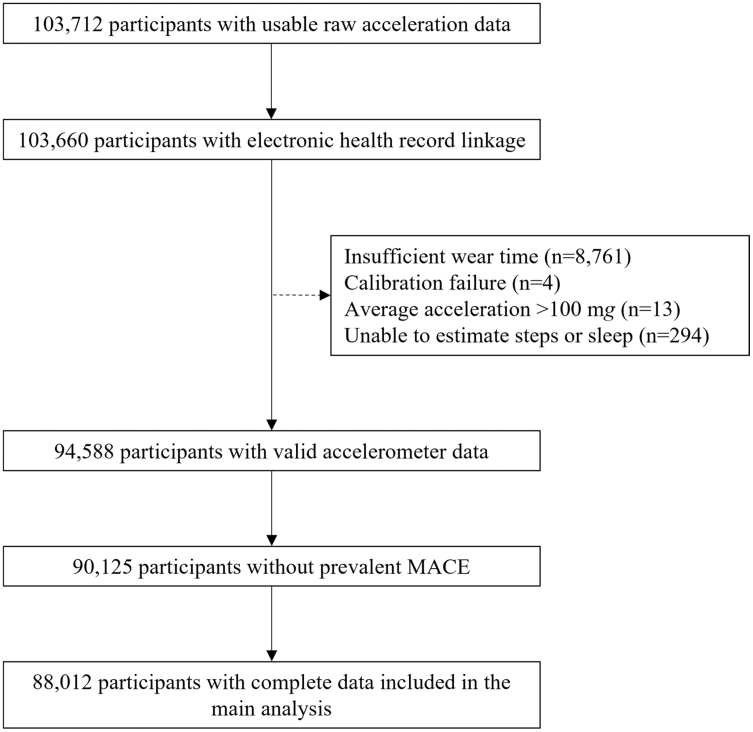
Flow diagram for participants included in the analysis of the independent and joint associations of accelerometer-measured step count and sleep duration with incident major adverse cardiovascular events (MACE).

Of the 103,712 participants with useable raw acceleration data, 52 were excluded due to withdrawal or loss of linked electronic health record follow-up before the accelerometry sub-study. Among the remaining 103,660, exclusions included 9072 for invalid accelerometer data, 4463 for prior MACE, and 2113 for missing covariates, resulting in a final analytic sample of 88,012 participants (Fig. 1). Participants excluded from the final analytic sample were, on average, slightly older and more likely to be male and obese (Table S8).

Table 1 presents the baseline characteristics of the final analytic sample, grouped by median daily step count. Among the participants, 58.0% (N: 51,046) were women, with a mean age of 62.2 years (SD: 7.8) at the end of accelerometer wear. Most were white (96.9%), from the least deprived TDI quintile (50.7%), and had a high level of education (43.9%). Compared with the high step count group, the low step count group had fewer participants with a high level of education, more current smokers, more obese participants, more long sleepers, and fewer short sleepers.

**Table 1:** Baseline characteristics of study participants by step count.

Characteristic	Median daily step count			Total
	Low (<7500)	Intermediate (7500–11,000)	High (11,000+)	
N	27,881	32,904	27,227	88,012
Female	16,523 (59.3%)	19,273 (58.6%)	15,250 (56.0%)	51,046 (58.0%)
Age at end of accelerometer wear (years)	62.8 (7.9)	62.2 (7.8)	61.4 (7.6)	62.2 (7.8)
Ethnicity				
White	26,918 (96.5%)	31,913 (97.0%)	26,449 (97.1%)	85,280 (96.9%)
Non-white	963 (3.5%)	991 (3.0%)	778 (2.9%)	2732 (3.1%)
Townsend Deprivation Index				
Least deprived	13,916 (49.9%)	17,050 (51.8%)	13,687 (50.3%)	44,653 (50.7%)
2nd quintile	6274 (22.5%)	7495 (22.8%)	6066 (22.3%)	19,835 (22.5%)
3rd quintile	3916 (14.0%)	4425 (13.4%)	3966 (14.6%)	12,307 (14.0%)
4th quintile	2769 (9.9%)	2982 (9.1%)	2643 (9.7%)	8394 (9.5%)
Most deprived	1006 (3.6%)	952 (2.9%)	865 (3.2%)	2823 (3.2%)
Highest education qualification				
School leaver	7299 (26.2%)	7385 (22.4%)	5436 (20.0%)	20,120 (22.9%)
Further education	9874 (35.4%)	10,734 (32.6%)	8686 (31.9%)	29,294 (33.3%)
Higher education	10,708 (38.4%)	14,785 (44.9%)	13,105 (48.1%)	38,598 (43.9%)
Smoking status				
Never	15,500 (55.6%)	19,339 (58.8%)	16,161 (59.4%)	51,000 (57.9%)
Previous	10,129 (36.3%)	11,510 (35.0%)	9493 (34.9%)	31,132 (35.4%)
Current	2252 (8.1%)	2055 (6.2%)	1573 (5.8%)	5880 (6.7%)
Alcohol intake				
Never	1867 (6.7%)	1654 (5.0%)	1317 (4.8%)	4838 (5.5%)
<3 times/week	13,995 (50.2%)	14,704 (44.7%)	11,325 (41.6%)	40,024 (45.5%)
3+ times/week	12,019 (43.1%)	16,546 (50.3%)	14,585 (53.6%)	43,150 (49.0%)
Processed meat intake				
<1 times/week	9100 (32.6%)	11,011 (33.5%)	8817 (32.4%)	28,928 (32.9%)
1–4 times/week	9977 (35.8%)	11,474 (34.9%)	9900 (36.4%)	31,351 (35.6%)
5+ times/week	8804 (31.6%)	10,419 (31.7%)	8510 (31.3%)	27,733 (31.5%)
Median overnight sleep duration				
Short (<6.5 h)	8837 (31.7%)	10,261 (31.2%)	9658 (35.5%)	28,756 (32.7%)
Intermediate (6.5–7.5 h)	11,411 (40.9%)	14,915 (45.3%)	12,239 (45.0%)	38,565 (43.8%)
Long (7.5+ hours)	7633 (27.4%)	7728 (23.5%)	5330 (19.6%)	20,691 (23.5%)
Body mass index (kg/m^2^)^a^				
Underweight (0–18.49)	104 (0.4%)	172 (0.6%)	193 (0.8%)	469 (0.6%)
Normal weight (18.5–24.9)	7576 (30.4%)	12,027 (40.7%)	11,626 (47.6%)	31,229 (39.6%)
Overweight (25–29.9)	10,139 (40.7%)	12,423 (42.1%)	9850 (40.3%)	32,412 (41.1%)
Obese (30+)	7090 (28.5%)	4913 (16.6%)	2753 (11.3%)	14,756 (18.7%)
Glycated haemoglobin (mmol/mol)^a^	36.0 (7.2)	35.2 (5.6)	35.0 (5.2)	35.4 (6.1)
Systolic blood pressure (mmHg)^a^	138.7 (18.7)	136.9 (18.6)	136.0 (18.4)	137.2 (18.6)
Diastolic blood pressure (mmHg)^a^	83.6 (10.4)	82.3 (10.4)	81.7 (10.3)	82.5 (10.4)
Total cholesterol (mmol/L)^a^	6.0 (1.1)	5.9 (1.0)	5.9 (1.0)	5.9 (1.0)

Results shown are mean (standard deviation) for normally distributed continuous variables, median (interquartile range) for non-normally distributed continuous variables, or number of participants (percentage) for categorical variables.

The median daily step count was 5896 for low step count, 9137 for intermediate step count, and 13,224 for high step count, with an overall median daily step count of 9105 for all participants.

a Results reported for a population of 78,866 participants after further exclusions due to missing data.

Among the 88,012 participants, 3817 cases of MACE were recorded over 680,723 person-years of follow-up (median follow-up: 7.9 years, IQR: 7.3–8.4). Of these cases, 57.1% (N: 2180) were non-fatal myocardial infarctions or strokes, 24.6% (N: 938) were revascularisation procedures, and 18.3% (N: 699) were cardiovascular deaths.

### Independent association of step count and incident major adverse cardiovascular events

**Fig. 2:**
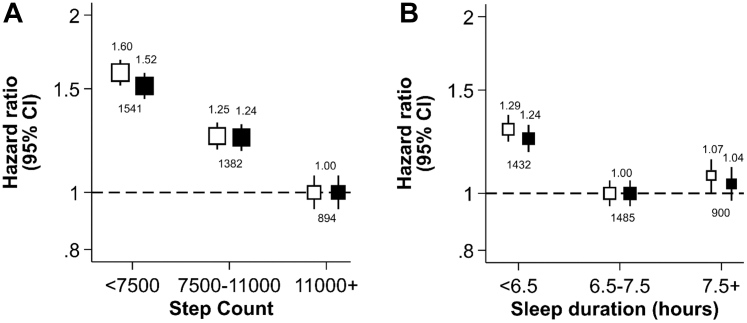
Independent associations of step count (A) and sleep duration (B) with incident major adverse cardiovascular events (MACE). HRs from both the minimally adjusted model (white boxes; adjusted for age and sex) and the maximally adjusted model (black boxes; additionally adjusted for ethnicity, education, TDI, smoking status, alcohol intake, processed meat intake, and sleep duration or step count) have been depicted for each participant group according to the following classifications for median daily step count (low [<7500], intermediate [7500–11,000], high [11,000+]) and median overnight sleep duration (short [<6.5 h], intermediate [6.5–7.5 h], long [7.5+ hours]). High step count and intermediate sleep duration were the reference groups. The vertical lines represent 95% CIs based on floating absolute risks. The size of each box is relative to the amount of statistical information available. Numbers above the boxes indicate the HRs, while numbers below indicate the total number of events in each group. The HRs for the y-axis have been plotted on a log scale. HR, hazard ratio; TDI, Townsend Deprivation Index; 95% CI, 95% confidence interval.

Fewer daily steps were associated with a higher risk of MACE (Fig. 2A). In the maximally adjusted model, participants in the low step count group had a 52% higher risk (HR: 1.52, 95% CI: 1.40–1.65, *p* < 0.0001; Figure S4A), and those in the intermediate group had a 24% higher risk (HR: 1.24, 95% CI: 1.14–1.35, *p* < 0.0001; Figure S4B), compared with the high step count reference group. BMI modestly attenuated the associations between step count and incident MACE (low: HR 1.42, 95% CI 1.30–1.55, *p* < 0.0001; intermediate: HR 1.21, 95% CI 1.11–1.32, *p* < 0.0001), whereas HbA1c, blood pressure, and total cholesterol had minimal impact (Figures S5A and S5B).

Excluding MACE cases within the first two, four, and six years progressively attenuated the associations (Figures S6A and S6B). After excluding the first six years, the low step count group had a 22% higher risk than the reference group (HR: 1.22, 95% CI: 1.05–1.42, *p* = 0.0095), while the association for the intermediate group was no longer suggestive of statistical significance (HR: 1.14, 95% CI: 0.98–1.32, *p* = 0.10). Excluding those with prior CVD or cancer attenuated the association for low step count (HR: 1.35, 95% CI: 1.18–1.54, *p* < 0.0001; Figure S7A), but not for intermediate step count (Figure S7B). Treating non-CVD death as a competing risk did not substantially alter the associations (Figure S8A and S8B). Excluding participants with factors that could disrupt sleep did not substantially alter the associations (Figures S9A and S9B). Adjustment for step cadence, but not sleep efficiency, attenuated the associations (Figures S10A and S10B). Finally, adjustment for both sedentary time and time spent in moderate-to-vigorous physical activity attenuated the associations (Figure S11A and S11B).

### Independent association of sleep duration and incident major adverse cardiovascular events

Short sleep duration was associated with a higher risk of MACE (Fig. 2B). In the maximally adjusted model, participants with short sleep duration had a 24% higher risk compared with the intermediate sleep duration reference group (HR: 1.24, 95% CI: 1.15–1.33, *p* < 0.0001; Figure S4C). In contrast, the risk of MACE among participants with long sleep duration was not significantly different from the reference group (HR: 1.04, 95% CI: 0.96–1.13, *p* = 0.38); Figure S4D). BMI modestly attenuated the association for short sleep duration (HR: 1.21, 95% CI: 1.12–1.30, *p* < 0.0001), whereas HbA1c, blood pressure, and total cholesterol had minimal impact (Figure S5C). The non-significant association for long sleep duration persisted across all mediator adjustments (Figure S5D).

Excluding participants with prior CVD or cancer fully attenuated the association between short sleep duration and incident MACE (HR: 1.10, 95% CI: 0.97–1.23, *p* = 0.13; Figure S7C). The other sensitivity analyses did not substantially alter the risk estimate (Figures S6C and S8C–S11C). The non-significant association for long sleep duration persisted across all sensitivity analyses (Figures S6D-S11D).

### Joint associations of step count and sleep duration with incident major adverse cardiovascular events

**Fig. 3:**
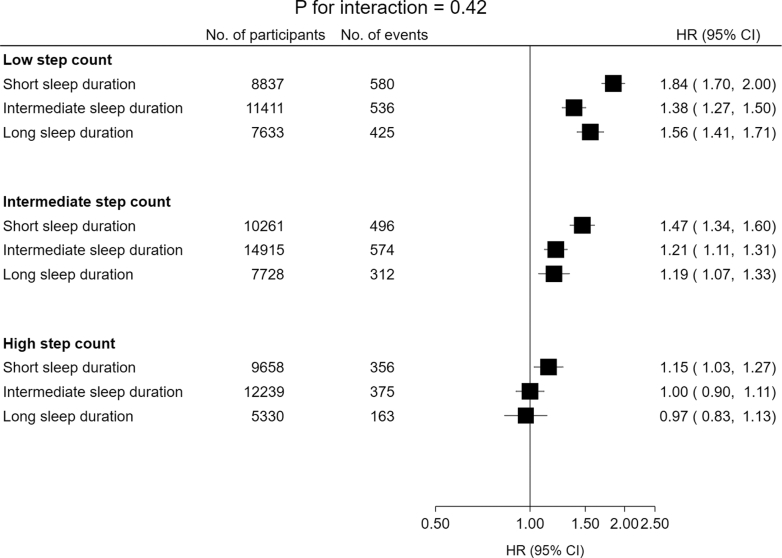
Joint associations of step count and sleep duration with incident major adverse cardiovascular events (MACE). Participants were stratified into nine mutually exclusive groups using the following classifications for median daily step count (low [<7500], intermediate [7500–11,000], high [11,000+]) and median overnight sleep duration (short [<6.5 h], intermediate [6.5–7.5 h], long [7.5+ hours]). Those with high step count and intermediate sleep duration served as the reference group. HRs from the maximally adjusted model (adjusted for age, sex, ethnicity, education, TDI, smoking status, alcohol intake, and processed meat intake) have been depicted for each participant group. The *P* for interaction was derived from a likelihood ratio test comparing the model with and without an interaction term between step count and sleep duration. The horizontal lines represent 95% CIs based on floating absolute risks, allowing direct comparisons between groups. The size of each box is relative to the amount of statistical information available. The HRs for the x-axis have been plotted on a log scale. HR, hazard ratio; TDI, Townsend Deprivation Index; 95% CI, 95% confidence interval.

Overall, there was no evidence of an interaction between step count and sleep duration with incident MACE (*P* for interaction = 0.42). Compared with the reference group—high step count and intermediate sleep duration—all other groups had a higher risk of MACE (Fig. 3 and Table S6), except participants with high step count and long sleep duration (HR: 0.97, 95% CI: 0.83–1.13, *p* = 0.74). The highest risk was among those with both low step count and short sleep duration (HR: 1.84, 95% CI: 1.70–2.00, *p* < 0.0001). Including all potential mediators (BMI, HbA1c, blood pressure, and total cholesterol) did not substantially alter the interaction or joint risk estimates (*P* for interaction = 0.52; Table S6 and Figure S12).

Excluding participants with prior CVD or cancer substantially attenuated the joint risk estimates, with only a few combinations remaining associated with a higher risk of MACE—specifically, intermediate step count with short sleep duration, and low step count with either short or long sleep duration (Table S6 and Figure S13). Excluding MACE cases within the first four years modestly attenuated the risk estimates (Table S6 and Figure S14). For example, participants in the least favourable group (low step count and short sleep duration) had a 67% higher risk of MACE compared with the reference group (HR: 1.67, 95% CI: 1.50–1.87, *p* < 0.0001). Treating non-CVD death as a competing risk did not substantially alter the risk estimates (Table S6 and Figure S15). Adjusting for step cadence and sleep efficiency (Table S6 and Figure S16) and adjusting for sedentary time and time spent in moderate-to-vigorous physical activity (Table S6 and Figure S17) modestly attenuated the risk estimates. In contrast, excluding participants with sleep-disrupting factors (Table S6 and Figure S18) did not substantially alter the risk estimates. Across all sensitivity analyses, there was no evidence of an interaction between step count and sleep duration with incident MACE.

## Discussion

In this study of over 88,000 UK Biobank participants with accelerometer data, we found no evidence of an interaction between step count and sleep duration with incident MACE. However, there was a strong independent inverse association between step count and incident MACE, with participants in the low step count group having a ∼40% higher risk compared with those in the high step count group. We also observed an independent L-shaped association between sleep duration and incident MACE, with participants in the short sleep duration group having a ∼20% higher risk compared with those with intermediate sleep duration. These findings highlight the importance of maintaining sufficient levels of both daily steps and sleep for the prevention of CVD, which could help inform future public health guidelines.

The lack of interaction in this study contrasts with findings from some previous studies using both self-reported and device-based data. For example, using self-reported data from over 340,000 participants in Taiwan, Chen et al. reported that those with low PA and long sleep duration had a 66% higher risk of CVD mortality compared with the reference group—participants with high PA and intermediate sleep duration (HR: 1.66, 95% CI: 1.43–1.92). They also found that high PA fully attenuated the elevated risk associated with long sleep duration (HR: 1.02, 95% CI: 0.81–1.29).^6^ In contrast, we found no association between long sleep duration and incident MACE. As Chen et al.’s study relied solely on self-reported measures, misclassification bias may have contributed to this discrepancy. For example, participants with poorer cardiovascular health may have overestimated their sleep duration.^26^

Liang et al. also reported an interaction between device-based PA and sleep duration with the risk of CVD mortality. Participants with both low PA and short sleep duration had nearly a four-fold higher risk than those with high PA and intermediate sleep duration (HR: 3.93, 95% CI: 2.90–5.32), and higher PA fully attenuated the elevated risk associated with short sleep duration (HR: 1.40, 95% CI: 0.88–2.25).^7^ We found no evidence of an interaction between step count and sleep duration with incident MACE. However, similar to Liang et al., we observed that participants with both low step count and short sleep duration had the highest risk of MACE, although the association was weaker in our study (HR: 1.84, 95% CI: 1.70–2.00). This difference likely reflects our inclusion of non-fatal CVD events and revascularisation procedures, while more severe outcomes such as CVD mortality tend to show stronger associations with PA and sleep. However, our broader MACE definition resulted in more cases (N: 3817) than Liang et al. (N: 1074), reducing random error—an important consideration when stratifying by multiple groups to examine interaction effects.

The independent association between step count and incident MACE in our study aligns with the existing literature. For example, a meta-analysis by Stens et al., using device-based step count data from over 85,000 participants across four prospective cohort studies, found that those in the low step count group had more than twice the risk of CVD compared with the high step count group (HR: 2.38, 95% CI: 1.88–3.03).^22^ Our more conservative estimate may reflect the higher median daily step count in our low step count group (5896 steps) compared with theirs (2022 steps), as lower step counts tend to show stronger associations with incident CVD. More daily steps may benefit the cardiovascular system through multiple pathways, including lowering total cholesterol and lipoproteins, reducing systemic inflammation and ambulatory blood pressure, and improving insulin receptor sensitivity.^27^

Our findings on the independent association between sleep duration and incident MACE differ from those of previous studies. For example, a large meta-analysis of over three million participants across 74 observational studies reported a J-shaped association, with both short (<6 h) and long (>9 h) sleep durations associated with a higher risk of CVD mortality compared with intermediate sleep duration (7 h).^19^ In contrast, we found that only short sleep duration was associated with a higher risk of MACE. Besides misclassification bias from self-reported sleep duration, this discrepancy may stem from some of the meta-analysis studies not excluding participants with latent or prevalent CVD, potentially introducing reverse causation bias where long sleep duration reflects underlying disease.^28^ Insufficient sleep has been suggested to contribute to atherosclerosis through immune dysregulation, upregulation of the sympathetic nervous system, and endocrine hormone imbalances.^29^

Our findings suggest reverse causality may influence both the independent and joint associations of step count and sleep duration with incident MACE. Excluding the first six years of follow-up notably attenuated the risks associated with fewer daily steps, but not short sleep duration, possibly because latent CVD more strongly affects PA than sleep duration.^26^^,^^30^ However, excluding participants with prior CVD or cancer attenuated the associations for both exposures, likely due to the removal of participants with more chronic conditions, such as heart failure, which impact both PA and sleep duration.^28^ Nevertheless, although adjustment for prior disease weakened the associations, they largely remained statistically significant, supporting the robustness of our main findings.

Strengths of our study include the large number of MACE events, which provided sufficient statistical power even after categorising participants into nine groups, as well as the small proportion of missing covariate data (∼2.0%) and loss to follow-up (<0.01%). We acknowledge that different types of MACE may exhibit distinct associations with step count and sleep duration; however, we focused on overall MACE to maximise events and statistical power. Future research should examine associations with specific MACE types to explore potential differential effects.

We used open-source machine learning algorithms to derive step count and sleep duration from raw acceleration data, which have shown greater reliability and validity than self-reported measures.^16^^,^^17^ PA was assessed using step count—a clinically interpretable and widely adopted metric^20^—enhancing the applicability of our findings to health promotion strategies. We also adjusted for a broad range of key covariates, allowing us to assess their potential confounding and mediating effects on the observed associations. To explore potential mediation, we conducted stepwise adjustment by sequentially including covariates (BMI, HbA1c, blood pressure, and total cholesterol) to examine how the associations between the exposures and outcome were attenuated. These analyses are descriptive and should not be interpreted as formal evidence of mediation; collider bias cannot be ruled out.

Several limitations of our study should be acknowledged. The measurement period was limited to a maximum of seven days per participant, which may not fully capture habitual PA and sleep patterns. This could lead to regression dilution bias and an underestimation of the true associations.^31^ Most covariates were measured at study entry, several years before accelerometer wear, potentially introducing measurement error. However, previous UK Biobank analyses suggest that key covariates are generally stable over time, somewhat mitigating this concern.^32^ As with all observational studies, despite adjustment for key confounders across demographic, socioeconomic, and lifestyle domains, residual confounding remains possible.

The external validity of our findings may be limited because UK Biobank participants are predominantly white (97% in the current analytic sample) and have been shown to be more physically active, wealthier, and healthier than the general population.^33^ The baseline characteristics of participants excluded from the final analytic sample also differed from those included—particularly in terms of sex, age, and BMI—which may further affect the generalisability of our findings.

Very low step counts and very long sleep durations were rare in our sample, limiting statistical power to examine their associations with incident MACE. For this reason, we used a three-stage, nine-cell grid to visualise how step count and sleep duration are jointly associated with incident MACE. In future studies with more MACE events, it may be possible to quantify the “optimal zone”, representing the range of step counts and sleep durations associated with the lowest risk.

In summary, fewer daily steps and short sleep duration were independently associated with a higher risk of MACE, with no evidence of an interaction between the two. Further research using repeated, longer-term measurements, extended follow-up to mitigate reverse causation, and replication in other cohorts is warranted.

## Contributors

Concept and design: JY, CH, and AD. Data access and verification: JY and CH. Statistical analysis: JY and LB. Data interpretation: all authors. Drafting of the manuscript: LB and JY. Critical revision of the manuscript: all authors. All authors had full access to all the data in the study and had final responsibility for the decision to submit for publication.

## Data sharing statement

Data may be obtained from a third party and are not publicly available. This research was conducted using the UK Biobank Resource under Application Number 59070. Data are accessible through the UK Biobank following an application process and approval from the UK Biobank Research Ethics Committee.

## Declaration of interests

AD is funded by grants from the Wellcome Trust (223100/Z/21/Z and 227093/Z/23/Z); Novo Nordisk; Swiss Re; Boehringer Ingelheim; the British Heart Foundation Centre of Research Excellence (RE/18/3/34214); and Health Data Research UK, which is funded by UK Research and Innovation and the UK Department of Health and Social Care. AD also receives research funding and software-licence royalties from GlaxoSmithKline; receives financial donations, paid to his institution, to purchase equipment from Swiss Re; funding from Google to support research into wearables phenotyping; donations of equipment from Google; receives honoraria as a grant panel member for the Wellcome Trust, UK Research and Innovation, and the UK National Institute for Health and Care Research; receives personal payments from Harvard University as a US National Institutes of Health grant, the University of Wisconsin as a US National Institutes of Health grant, and the Wellcome Trust; receives honoraria from the American Academy of Insurance Medicine for thesis examinations; receives financial support to attend the 2024 International consensus meeting on mobility measurement, the 2023 Wellcome Trust and Google workshop on mental health, the 2023 Big Model AI for Drug Design workshop, the 2020 3rd New York University Biomedical and Biosystems Conference, the 2020 Academy of Medical Sciences UK–Japan Symposium on Data-Driven Health, the 2019 SLEEP Meeting, and the 2017 Hong Kong Academy of Medicine Annual Scientific Meeting; and is on scientific advisory boards for the EU iPROLEPSIS project on psoriatic arthritis inflammation and the EU IMI IDEA-FAST project on wearable sensors in neurodegenerative trials.
